# Efficacy and Safety of Dulaglutide Biosimilar LY05008 Versus the Reference Product Dulaglutide (Trulicity) in Chinese Adults With Type 2 Diabetes Mellitus: A Randomized, Open‐Label, Active Comparator Study

**DOI:** 10.1111/1753-0407.70077

**Published:** 2025-04-11

**Authors:** Li Liu, Zhifeng Cheng, Lianwei Wang, Lili Zhang, Shunbin Li, Shu Li, Shuguang Pang, Qifu Li, Fang Bian, Junling Gu, Jie Shen, Liujun Fu, Baiping Sun, Yanyan Zhao, Changlin Dou, Zhaoyang Zeng, Lixin Guo

**Affiliations:** ^1^ Beijing Hospital Beijing China; ^2^ The Fourth Affiliated Hospital of Harbin Medical University Harbin Heilongjiang China; ^3^ Zhumadian Central Hospital Zhumadia Henan China; ^4^ Jincheng Hospital Jinchen Shanxi China; ^5^ Huzhou Central Hospital Zhejiang China; ^6^ Huizhou Central Hospital Huizhou Guangdong China; ^7^ Jinan Central Hospital Jinan City Shandong China; ^8^ The First Affiliated Hospital of Chongqing Medical University Chongqing China; ^9^ People's Hospital of Cangzhou Cangzhou Hebei China; ^10^ Second People's Hospital of Yibin Yibin Sichuan China; ^11^ Shunde Hospital of Southern Medical University Foshan Guangdong China; ^12^ The First Affiliated Hospital of Henan University of Science and Technology Luoyang Henan China; ^13^ Shandong Boan Biotechnology Co. Ltd Yantai China; ^14^ Yichang Central People's Hospital Yichang Hubei China

**Keywords:** biosimilar, dulaglutide, HbA1c, safety

## Abstract

**Background:**

Dulaglutide, a glucagon‐like peptide‐1 (GLP‐1) receptor agonist, has been approved for improving glycemic control and reducing the risk of cardiovascular (CV) adverse events. A previous result in healthy Chinese male subjects demonstrated the pharmacokinetic (PK) similarity of LY05008 and the licensed product dulaglutide, with comparable safety and immunogenicity profiles. A well‐controlled phase 3 study with an adequate sample size was subsequently conducted for safety and efficacy evaluation.

**Methods:**

In a multicenter, randomized, open‐label, active comparator phase 3 study, Chinese adults diagnosed with type 2 diabetes mellitus (T2DM) were randomly assigned 1:1 to receive a subcutaneous injection of 1.5 mg LY05008 or dulaglutide once weekly for 24 weeks. The primary endpoint was the mean change in HbA1c from baseline to Week 24. The secondary endpoints included the mean change in HbA1c from baseline to Week 12; the proportion of patients who had achieved HbA1c ≤ 6.5% at Weeks 12 and 24; and the mean change in body weight, fasting plasma glucose (FPG) level, and 2‐h postprandial plasma glucose (PPG) level from baseline to Weeks 12 and 24. Safety, PK, and immunogenicity profiles were also included for data analysis.

**Results:**

A total of 440 patients were randomized to receive LY05008 (*n* = 222) or dulaglutide (*n* = 218). The mean changes in HbA1c from baseline to Week 24 in the LY05008 group and dulaglutide group were −1.44% and −1.41%, respectively, with a least square mean difference (LSMD) and 95% confidence interval (CI) of 0.06% (−0.08, 0.19) (*p* > 0.05). Efficacy equivalence could be demonstrated since the 95% CI between the reference drug and a biosimilar fell entirely within the range of (−0.4%, 0.4%). The mean changes in HbA1c from baseline to Week 12 in the LY05008 group and dulaglutide group were −1.47% and −1.39% (*p* > 0.05), respectively. At Week 12, 40.1% of patients who received LY05008 and 42.2% of those who received dulaglutide had a decrease in the HbA1c level to 6.5% or less, and 60.4% and 60.6% of patients in the LY05008 group and the dulaglutide group had a decrease in the HbA1C level < 7%, respectively. At Week 24, 41.0% and 43.6% of patients achieved an HbA1c ≤ 6.5%. 55.9% and 66.5% of patients in the LY05008 group and the dulaglutide group achieved the HbA1c goal of < 7%, respectively. The mean changes in body weight from baseline to Weeks 12 and 24 in the LY05008 group and dulaglutide group were −2.01 and −1.71 kg (*p* > 0.05) and −2.68 and −2.42 kg (*p* > 0.05), respectively. The mean changes in FPG level from baseline to Weeks 12 and 24 in the LY05008 group and dulaglutide group were −2.578 and −2.681 mmol/L (*p* > 0.05) and −2.222 and −2.690 mmol/L, respectively. In the LY05008 group and the dulaglutide group, the mean changes in 2‐h PPG levels from baseline to Weeks 12 and 24 were −4.364 and −4.800 mmol/L(*p* > 0.05) and−3.502 and −4.217 mmol/L (*p* > 0.05), respectively. The common treatment emergent adverse events (TEAEs) in the LY05008 and dulaglutide groups were decreased appetite, diarrhea, upper respiratory tract infection, hyperuricemia, nausea, urinary tract infection, and vomiting. Most TEAEs were mild to moderate in severity. No significant differences were observed between the groups in terms of TEAEs. Hypoglycemic events were noted in 0.9% of patients who had received LY05008 and in 3.7% of those who had received dulaglutide. Serious adverse events were reported in 4.1% of patients in the LY05008 group and in 3.7% of patients in the dulaglutide group. The PK parameter C_trough_ and immunogenicity profiles were similar across the two treatment groups.

**Conclusion:**

The primary endpoint was met in this study through the demonstration of equivalent efficacy in HbA1c reduction in Chinese adults with T2DM between LY05008 and dulaglutide. Overall, the biosimilar product LY05008 showed comparable safety, PK, and immunogenicity profiles against the reference drug dulaglutide.

**Trial Registration:** ClinicalTrials.gov identifier: CTR20221721

## Graphical abstract

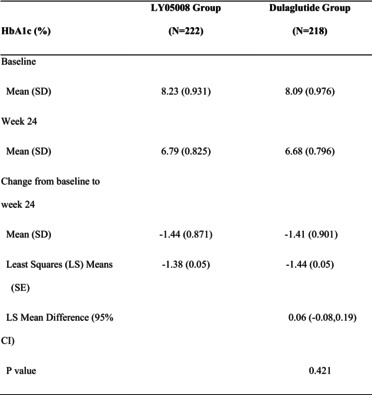

**Summary**

- Patients with type 2 diabetes mellitus could experience acute and chronic complications, which lead to impaired functional status and quality of life.
- The reference drug, dulaglutide (Trulicity) is indicated for the management of patients with type 2 diabetes mellitus.
- LY05008, dulaglutide biosimilar, demonstrated equivalent efficacy in HbA1C reduction in Chinese adults with type 2 diabetes mellitus when compared with the reference drug.

## 1 Introduction

Approximately 529 million people are living with diabetes globally, and the prevalence of global age‐standardized total diabetes, primarily T2DM, was 6.1% in 2021 [1]. T2DM is largely attributed to a high body mass index (BMI) and has led to a 24.3% increase in disability‐adjusted life‐years between 1990 and 2021 [1]. It is estimated that more than 1.31 billion people will have T2DM worldwide by 2050 [1].

Per American Diabetes Association‐Standard of Care (ADA SOC) 2024, the drug class, known as GLP‐1 receptor agonist, is considered as very high efficacy medication for glucose lowering and weight loss [2]. Meanwhile, it provides additional benefits to reduce major adverse cardiovascular events in T2DM patients who have established cardiovascular disease (CVD) or those who are at increased risk factors for CVD [2]. Dulaglutide is an incretin mimetic with 90% amino acid sequence homology to endogenous human GLP‐1 [3]. It is a long‐acting GLP‐1 receptor agonist that triggers a downstream cell signaling pathway to elicit glucose‐dependent insulin secretion [4], delay gastric emptying, and promote satiety, which leads to weight loss [5]. The US Food and Drug Administration (FDA) issued the initial approval of dulaglutide for improving glycemic control in adults with T2DM in 2014 [6]. In 2019, dulaglutide was approved by the National Medical Products Administration (NMPA) for glycemic control by means of monotherapy plus diet and exercise or as an add‐on to other non‐GLP‐1 agents in the T2DM population [7].

Biosimilar products provide more opportunities for patients to access additional treatment options by reducing expenditures to a greater extent [8, 9]. Guidelines on scientific considerations for the development and evaluation of biosimilars were clearly established by the US FDA, EMA, NMPA, and World Health Organization (WHO) to ensure the standardization, reliability, and integrity of the development process [10, 11, 12, 13].

LY05008 is a dulaglutide biosimilar developed by Shandong Boan Biotechnology Co. Ltd. A previous phase 1 study demonstrated that the PK profiles of LY05008 were similar to dulaglutide in healthy Chinese male subjects following a single dose of 0.75 mg subcutaneous injection. Similar safety profiles of adverse events between the two treatment groups were noted. Non‐clinical and phase 1 results supported the further development and evaluation of LY05008 in patients with T2DM. The purpose of the phase 3 trial is to prove pharmaceutical equivalence between LY05008 and dulaglutide regarding efficacy, safety, PK, and immunogenicity in Chinese adults with T2DM who were inadequately controlled with metformin monotherapy.

## 2 Patients and Methods

### 2.1 Human Rights Statement

This multicenter clinical study was conducted in compliance with the provisions of the Declaration of Helsinki, the International Conference on Harmonization E6 Guidelines on Good Clinical Practice, and local regulatory requirements. Written informed consent was provided by all the patients prior to enrollment in the study. The final protocol, amendments, and informed consent documents were reviewed and approved by the Institutional Review Board of Beijing Hospital. The trial was registered at the Clinical Trial Registry (Identifier No. CTR20221721).

### 2.2 Study Population

The key inclusion criteria included patients aged 18–75 years who were diagnosed with T2DM for at least 6 months and those with inadequate blood glucose control following the use of a stable dose of metformin at 1.5 to 2.0 g daily for at least 8 weeks prior to screening. Eligible patients had BMIs ranging from 18.5 to 35 kg/m^2^ and HbA1c levels ranging from 7% to 11% at screening and baseline. The key exclusion criteria were type 1 diabetes; acute diabetic complications such as diabetic ketoacidosis and hyperglycemic hyperosmolar state; severe hypoglycemia; current pancreatitis or a history of chronic or acute pancreatitis; serum amylase levels ≥ 3 times the upper limit of normal at screening or baseline; an estimated glomerular filtration rate below 60 mL per minute per 1.73 m^2^; abnormal gastric emptying; any personal or family history of medullary thyroid C‐cell hyperplasia, focal hyperplasia, medullary thyroid carcinoma (MTC), or multiple endocrine neoplasia type 2 (MEN 2); and a history of any of the following within 6 months at screening or baseline: acute coronary syndrome, coronary artery bypass grafting, coronary intervention (excluding diagnostic angiography), congestive heart failure (New York Heart Association [NYHA] Class > II), serious arrhythmia or arrhythmia requiring urgent intervention, or cerebrovascular accident.

### 2.3 Study Design

This 28‐week, randomized, open‐label, active‐controlled phase 3 study was conducted at 39 clinical sites in China. All patients were processed to a 14‐day screening followed by a 2‐week lead‐in period, during which physical exercise and dietary control were guided and restricted. Eligible patients were enrolled and randomized in a 1:1 ratio to receive 1.5 mg LY05008 or dulaglutide subcutaneously once a week for a 24‐week treatment period. Metformin (1.5 to 2.0 g daily) was used as a background therapy by all the enrolled patients during the study. Patients were scheduled for visits during screening, baseline, at week 2, 4, 8, 12, 16, 20, and 24 following the dose administration, during which all the required laboratory tests and study procedures were strictly conducted. The evaluation of safety, efficacy, PK, and immunogenicity was clearly specified in the protocol.

### 2.4 Endpoints

#### 2.4.1 Efficacy Parameters

The primary endpoint was the mean change in HbA1c from baseline to Week 24. The secondary endpoints included the mean change in HbA1c from baseline to Week 12; the proportion of patients who achieved an HbA1c ≤ 6.5% or < 7% at Weeks 12 and 24; and the mean change in body weight, fasting plasma glucose (FPG) level, and 2‐h postprandial plasma glucose (PPG) level from baseline to Weeks 12 and 24.

#### 2.4.2 Safety Parameters

The safety endpoints were any adverse events (AEs), including TEAE, treatment‐related adverse events (TRAEs), serious adverse events (SAEs), and so forth. Other safety endpoints included vital signs, physical examination results, laboratory test results, 12‐lead electrocardiogram (ECG) data, and the incidence of hypoglycemia. All AEs were assessed as mild, moderate, or severe in severity. All AEs were coded via the Medical Dictionary of Regulatory Activities (MedDRA) Version 26.1 and assessed by investigators for severity and relationship with the study drugs. All AEs were recorded and monitored throughout the study.

#### 2.4.3 PK Analysis

The PK parameter C_trough_ was analyzed using Phoenix WinNonlin (WNL) Version 7.0 or higher and summarized via descriptive statistics. Blood samples for C_trough_ were collected at D1, D29, D57, D85, D113, D141, and D169 prior to drug administration. Three milliliters of whole blood were collected at each time point, and the plasma concentrations of LY05008 and dulaglutide were measured and analyzed.

#### 2.4.4 Immunogenicity Analysis

Blood samples were collected for immunogenicity evaluation at D1, D29, D57, D85, D113, D141, and D169 prior to drug administration. Four milliliters of whole blood were collected at each time point to detect anti‐drug antibody (ADA) and neutralizing antibody (Nab) positivity. The ADA‐positive samples were further tested by a Meso Scale Diagnostics (MSD) Bridging‐Electrochemiluminescent immunoassay (ECLIA) for titer and Nab analysis.

### 2.5 Statistical Analysis

The study was designed to demonstrate the equivalence between 1.5 mg LY05008 and the matching dose of dulaglutide with respect to the change from baseline in HbA1c level at Week 24, with an equivalence margin of 0.4% [14]. An estimated 440 patients would be sufficient to provide 85% power to show equivalence at a two one‐sided type I error rate of 0.025, assuming a standard deviation of 1.1 and a dropout rate of 20%. All the statistical analyses were conducted via SAS Version 9.4 or higher.

For the primary and secondary efficacy analyses, the analysis of covariance (ANCOVA) was performed on the full analysis set (FAS). The model included the change in HbA1c level from baseline to Week 24 as the dependent variable and the baseline HbA1c level, stratification factors (sex, HbA1c level < 8.5% and ≥ 8.5%) and group as independent variables. The efficacy of the two products was considered similar if the 95% confidence interval of the least square mean (LSM) difference in the change from baseline in HbA1c level to Week 24 between the LY05008 group and the dulaglutide group was within the −0.4% to 0.4% range.

The safety set (SS) and pharmacokinetic concentration set (PKCS) were used for safety, C_trough_, and immunogenicity evaluation.

## 3 Results

### 3.1 Patient Disposition and Baseline Characteristics

**FIGURE 1:**
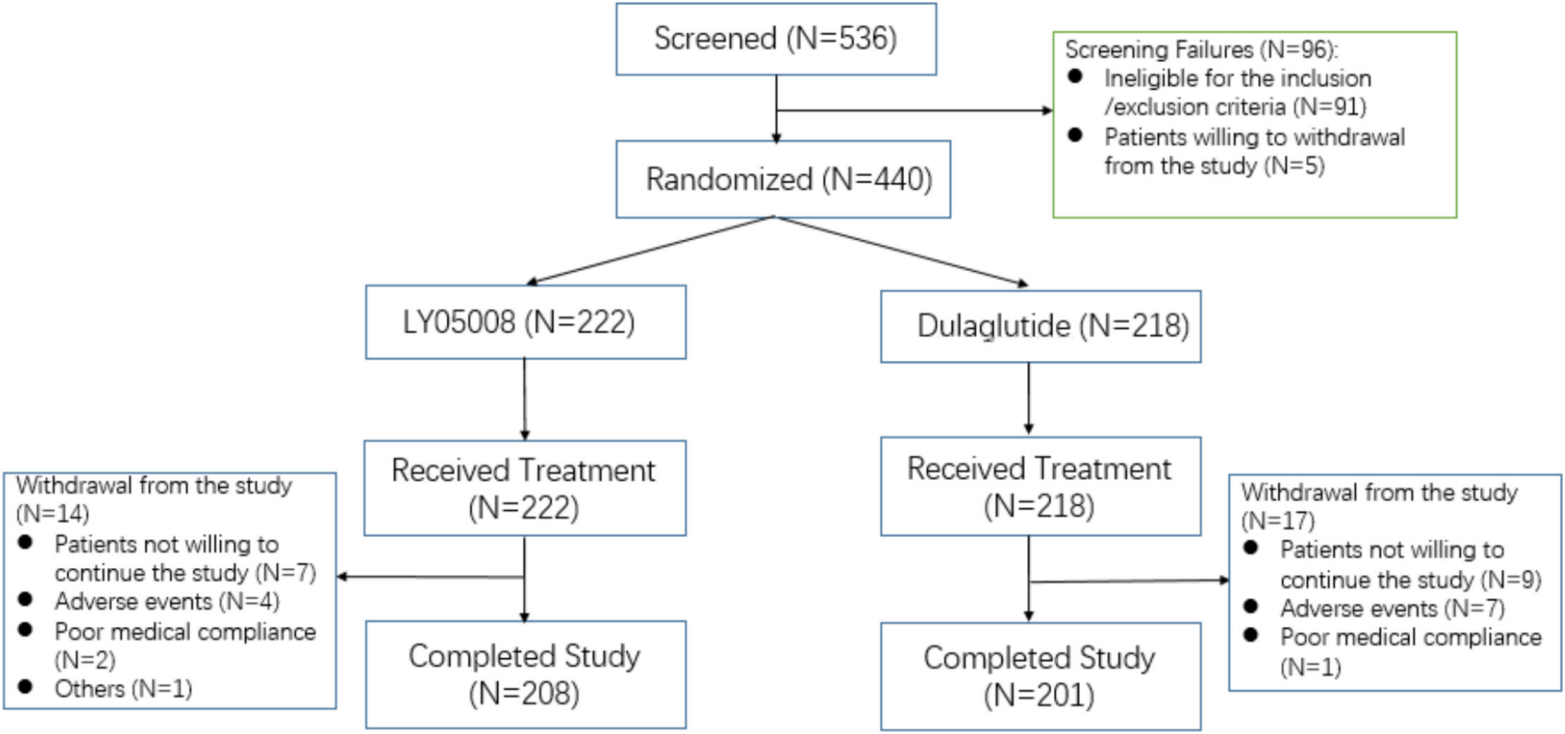
Flow chart of patient allocation. *N* indicates the number of patients.

A total of 536 patients with T2DM were screened and met the qualifications to advance into the enrollment process. A 420 (95.5%) patients were included in the per protocol set (PPS) with 213 (95.9%) patients in the LY05008 group and 207 (95.0%) patients in the dulaglutide group. A total of 440 eligible patients included in the FAS and SS were enrolled and randomized at a 1:1 ratio to one of the two treatment groups, with 222 patients in the LY05008 group and 218 patients in the dulaglutide group. All patients in both groups received a subcutaneous injection of 1.5 mg LY05008 or dulaglutide once weekly. A total of 208 patients in the LY05008 group completed the study, whereas 201 patients in the dulaglutide group completed the study. A total of 31 patients (14 patients in the LY05008 group and 17 patients in the dulaglutide group) withdrew from the study because they were unwilling to continue the study or because of AEs, medical compliance, and other factors. The number of patients who completed the study and the discontinuation rate were comparable between the two treatment groups. Patient disposition is summarized for all screened patients in Figure 1.

In the FAS, most T2DM patients included in the study were male Chinese (59.1%), with similar proportions of enrollment in either group. The mean age (SD), weight (SD) and BMI (SD) in the LY05008 group were 52.9 (11.39) years, 73.40 (11.691) kg, and 26.687 (3.1529) kg/m^2^, respectively. In the dulaglutide group, the mean age (SD), height (SD), weight (SD), and BMI (SD) were 52.0 (11.31) years, 165.53 (8.743) cm, 73.94 (13.380) kg, and 26.832 (3.3683) kg/m^2^, respectively. 249 (56.5%) and 191 (43.4%) patients had the baseline HbA1C level < 8.5% or ≥ 8.5%. In the LY05008 group, 55.9% of patients had a baseline HbA1c level < 8.5%, and 44.1% of patients had a baseline HbA1C level ≥ 8.5%. Similarly, 57.3% and 42.7% of patients with HbA1c levels < 8.5% and ≥ 8.5%, respectively, were in the dulaglutide group. The mean (SD) disease course of T2DM patients in the LY05008 group and dulaglutide group was 7.344 (5.0769) years and 7.173 (5.1294) years, respectively. No significant differences in baseline characteristics were observed between the two groups.

In the FAS, 169 patients in the LY05008 group and 162 patients in the dulaglutide group had received at least one concomitant medication during the study. The most commonly used concomitant medications according to the Anatomical Therapeutic Chemical (ATC) classification system in the LY05008 group and dulaglutide group were lipid‐modifying agents (26.6% vs. 24.8%), angiotensin converting enzyme inhibitors (22.5% vs. 23.9%) and calcium channel blockers (24.3% vs. 19.3%). There was a similar proportion of patients taking concomitant medications in the two treatment groups.

For the SS, the mean treatment compliance (SD) rates of the LY05008 and dulaglutide groups were 97.88 (7.610) % and 96.88 (11.727) %, respectively, which represented similar proportions between the two groups. The mean treatment compliance rates for daily metformin use were comparable between the LY05008 group (99.01%) and the dulaglutide group (98.78%) regardless of the lead‐in or treatment period.

All patients were analyzed for baseline characteristics per FAS (except for treatment compliance), and the parameters listed were comparable between the LY05008 group and the dulaglutide group (Table 1).

**TABLE 1:** Demographics and baseline characteristics—FAS.

Parameters	LY05008 group (*N* = 222)	Dulaglutide group (*N* = 218)	Total (*N* = 440)
Age (years)			
Mean (SD)	52.9 (11.39)	52.0 (11.31)	52.4 (11.34)
Sex			
Male, *n* (%)	132 (59.5)	128 (58.7)	260 (59.1)
Female, *n* (%)	90 (40.5)	90 (41.3)	180 (40.9)
Ethnicity			
Han, *n* (%)	216 (97.3)	208 (95.4)	424 (96.4)
Others, *n* (%)	6 (2.7)	10 (4.6)	16 (3.6)
Height (cm)			
Mean (SD)	165.57 (8.256)	165.53 (8.743)	165.55 (8.491)
Weight (kg)			
Mean (SD)	73.40 (11.691)	73.94 (13.380)	73.66 (12.545)
BMI (kg/m^2^)			
Mean (SD)	26.687 (3.1529)	26.832 (3.3683)	26.759 (3.2585)
Course of disease (years)			
Mean (SD)	7.344 (5.0769)	7.173 (5.1294)	7.259 (5.0979)
HbA1c %			
< 8.5%, *n* (%)	124 (55.9)	125 (57.3)	249 (56.6)
≥ 8.5%, *n* (%)	98 (44.1)	93 (42.7)	191 (43.4)
Prior metformin taken/day(g)^a^			
Mean (SD)	1.64 (0.223)	1.67 (0.236)	1.65 (0.229)
CrCL (mL/min)			
Mean (SD)	126.59 (43.329)	123.26 (42.803)	124.94 (43.052)

*Note:* Percentage is computed based upon the number of total patients in the given treatment group.

Abbreviations: BMI = body mass index; CrCL = creatinine clearance; FAS = full analysis set; SD = standard deviation.

^a^ Stable dose taken for at least 8 weeks or more prior to the screening date.

### 3.2 Efficacy

#### 3.2.1 Glycemic Endpoints

**FIGURE 2:**
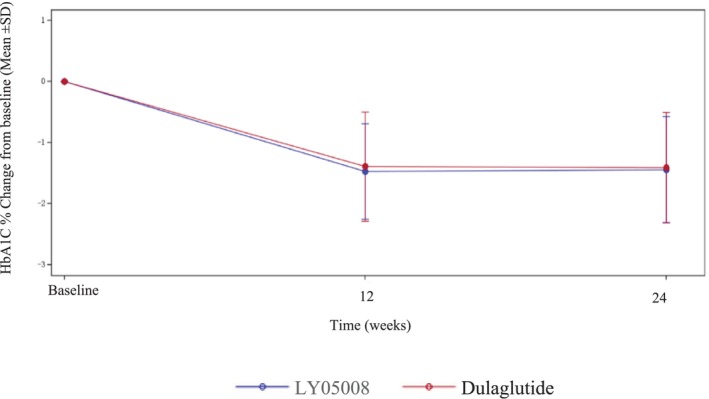
Mean changes in the HbA1c level (%) from baseline to week 24. Blue represents LY05008; pink represents dulaglutide.

For the primary endpoint, the reduction in the mean HbA1c level from baseline in the LY05008 group at Week 24 was −1.44%, whereas it was −1.41% in the dulaglutide group; these trends were similar between the two groups (Figure 2). The results from the ANCOVA model revealed that the LSM of the change in HbA1c level from baseline to Week 24 in the LY05008 group and dulaglutide group was −1.38% and −1.44%, respectively, with an LSMD of 0.06% (95% CI: −0.08, 0.19) (*p* > 0.05), which fell within the range of −0.4% to 0.4% (Table 2). The efficacy biosimilarity between LY05008 and dulaglutide was demonstrated since the primary endpoint was met. For the FAS, the LSM of the changes in the HbA1c level from baseline to Week 24 in the LY05008 group and dulaglutide group were −1.39% and −1.45%, respectively, with an LSMD of 0.06% (95% CI: −0.08, 0.19) (*p* > 0.05) according to the MMRM analysis. For PPS, the LSM of the changes in the HbA1c level from baseline to Week 24 in the LY05008 group and dulaglutide group were −1.38% and −1.45%, respectively, with an LSMD of 0.07% (95% CI: −0.06, 0.21) (*p* > 0.05) according to the ANCOVA (Table 3). The results obtained from the sensitivity analysis were congruent with those in the primary analysis. Moreover, the primary analysis was reassessed on the basis of the subgroup analysis to evaluate the consistency of efficacy between the different subgroups. The ANCOVA revealed that there was no statistically significant difference in clinical efficacy between the LY05008 group and the dulaglutide group (*p* > 0.05) (Table 4).

**TABLE 2:** Mean change from baseline in the HbA1c level (%) at week 24 via ANCOVA (FAS Population).

HbA1c (%)	LY05008 group (*N* = 222)	Dulaglutide group (*N* = 218)
Baseline		
Mean (SD)	8.23 (0.931)	8.09 (0.976)
Week 24		
Mean (SD)	6.79 (0.825)	6.68 (0.796)
Change from baseline to week 24		
Mean (SD)	−1.44 (0.871)	−1.41 (0.901)
Least squares (LS) Means (SE)	−1.38 (0.05)	−1.44 (0.05)
LS mean difference (95% CI)		0.06 (−0.08,0.19)
*p*		00.421

*Note:* (1) Baseline is defined as the last non‐missing value before the first‐dose administration; (2) descriptive analysis was based on the actual observed values; (3) statistical analysis was performed via ANCOVA with the change from baseline in the HbA1c level at week 24 as the dependent variable and the baseline HbA1c level, stratification factors (sex and HbA1c level [< 8.5% and ≥ 8.5%]) and the grouping as the independent variables; (4) if the difference in the 95% confidence interval between the groups falls within the range [−0.4%, 0.4%], the efficacy of LY05008 is equivalent to that of dulaglutide.

**TABLE 3:** Mean change from baseline in the HbA1c level (%) at week 24 via ANCOVA (PPS population).

HbA1c (%)	LY05008 group (*N* = 213)	Dulaglutide group (*N* = 207)
Baseline		
Mean (SD)	8.21 (0.937)	8.07 (0.984)
Week 24		
Mean (SD)	6.79 (0.826)	6.67 (0.789)
Change from baseline to week 24		
Mean (SD)	−1.43 (0.877)	−1.42 (0.900)
Least squares (LS) Means (SE)	−1.38 (0.05)	−1.45 (0.05)
LS mean difference (95% CI)		0.07 (−0.06,0.21)
*p*		0.288

*Note:* (1) Baseline is defined as the last non‐missing value before the first‐dose administration; (2) descriptive analysis was based on the actual observed values; (3) statistical analysis was performed via ANCOVA with the change from baseline in the HbA1c level at week 24 as the dependent variable and the baseline HbA1c level, stratification factors (sex and HbA1c level [< 8.5% and ≥ 8.5%]) and the grouping as the independent variables; (4) if the difference in the 95% confidence interval between the groups falls within the range [−0.4%, 0.4%], the efficacy of LY05008 is equivalent to that of dulaglutide.

**TABLE 4:** Mean change from baseline in the HbA1c level (%) at week 24 in the subgroup analysis via ANCOVA—FAS.

	HbA1C (%)	LY05008 group (*N* = 222)	Dulaglutide group (*N* = 218)
Stratification factor‐sex			
Male (*N* = 260)			
	Least squares (LS) means (SE)	−1.41 (0.07)	−1.50 (0.07)
	LS mean difference 95% CI		0.09 (−0.09,0.27)
	*p*		0.337
Female (*N* = 180)			
	Least squares (LS) means (SE)	−1.36 (0.07)	−1.37 (0.07)
	LS mean difference 95% CI		0.01 (−0.19,0.22)
	*p*		0.891
Stratification factor‐HbA1C level			
< 8.5% (*N* = 249)			
	Least squares (LS) means (SE)	−1.04 (0.06)	−1.06 (0.06)
	LS mean difference 95% CI		0.02 (−0.14,0.17)
	*p*		0.830
≥ 8.5% (*N* = 191)			
	Least squares (LS) means (SE)	−1.84 (0.08)	−1.93 (0.09)
	LS mean difference 95% CI		0.09 (−0.14,0.33)
	*p*		0.426

*Note:* (1) Baseline is defined as the last non‐missing value before the first‐dose administration; (2) descriptive and statistical analyses are based upon the actual observed values; (3) subgroup analysis 1: the statistical analysis was performed via ANCOVA with the change from baseline in the HbA1c level at week 24 as the dependent variable and the baseline HbA1c level, the stratification factor sex, and the grouping as the independent variables; (4) subgroup analysis 2: the statistical analysis was performed via ANCOVA with the change from baseline in the HbA1c level at week 24 as the dependent variable and the baseline HbA1c level, the stratification factor HbA1c level (< 8.5% and ≥ 8.5%) and grouping as the independent variables.

The secondary endpoints are summarized in Tables 5 **and** 6. The mean changes in the HbA1c level from baseline to Week 12 in the LY05008 group and dulaglutide group were −1.47% and −1.39%, respectively (*p* > 0.05). At Week 12, 40.1% of patients who received LY05008 and 42.2% of those who received dulaglutide had a decrease in the HbA1c level to 6.5% or less, and 60.4% and 60.6%, respectively, had a decrease in the HbA1C level < 7%. At Week 24, 41.0% and 43.6% of patients in the LY05008 group and the dulaglutide group achieved the HbA1c goal of 6.5% or less, and 55.9% and 66.5%, respectively, achieved the HbA1c goal of < 7%. The mean changes in the FPG level from baseline to Week 12 in the LY05008 group and dulaglutide group were −2.578 and −2.681 mmol/L, respectively (*p* > 0.05). The mean changes in the FPG level from baseline to Week 24 in the LY05008 and dulaglutide groups were−2.222 and −2.690 mmol/L, respectively (*p* < 0.05). In the LY05008 group and dulaglutide group, the mean changes in the 2‐h PPG level from baseline to Week 12 were −4.364 and−4.800 mmol/L, respectively (*p* > 0.05). At Week 24, the mean changes in the 2‐h PPG level from baseline in the LY05008 and dulaglutide groups were −3.502 and −4.217 mmol/L, respectively (*p* > 0.05).

**TABLE 5:** Proportion of patients with HbA1c goal achievement at Week 12 and Week 24‐FAS.

	LY05008 group (*N* = 222)	Dulaglutide group (*N* = 218)	Total (*N* = 440)
At Week 12			
HbA1C < 7%, *n* (%)	134 (60.4)	132 (60.6)	266 (60.5)
HbA1C ≥ 7%, *n* (%)	79 (35.6)	72 (33.0)	151 (34.3)
Clopper‐Pearson (95% CI)	(53.60%, 66.84%)	(53.73%, 67.08%)	
At Week 12			
HbA1C ≤ 6.5%, *n* (%)	89 (40.1)	92 (42.2)	181 (41.1)
HbA1C > 6.5%, *n* (%)	124 (55.9)	112 (51.4)	236 (53.6)
Clopper‐Pearson (95% CI)	(33.59%, 46.86%)	(35.56%, 49.06%)	
At Week 24			
HbA1C < 7%, *n* (%)	124 (55.9)	145 (66.5)	269 (61.1)
HbA1C ≥ 7%, *n* (%)	84 (37.8)	56 (25.7)	140 (31.8)
Clopper‐Pearson (95% CI)	(49.06%, 62.50%)	(59.83%, 72.74%)	
At Week 24			
HbA1C ≤ 6.5%, *n* (%)	91 (41.0)	95 (43.6)	186 (42.3)
HbA1C > 6.5%, *n* (%)	117 (52.7)	106 (48.6)	223 (50.7)

*Note: n* represents the number of patients whose HbA1c goal level was achieved; the descriptive and statistical analyses were based on the actual observed values.

**TABLE 6:** Mean changes from baseline in the secondary outcome measures via ANCOVA—FAS.

Variable	LS mean change from baseline (SE)		LS mean difference	*p*
	LY05008 group (*N* = 222)	Dulaglutide group (*N* = 218)	(95% CI)	
HbA1c (%)				
At Week 12	−1.43 (0.04)	−1.42 (0.04)	−0.0028 (−0.13, 0.12)	0.964
Body weight (kg)				
At Week 12	−2.03 (0.16)	−1.71 (0.17)	−0.31 (−0.77, 0.14)	0.172
At Week 24	−2.66 (0.20)	−2.39 (0.20)	−0.27 (−0.81, 0.27)	0.332
FPG (mmol/L)				
At Week 12	−2.57 (0.12)	−2.59 (0.12)	0.02 (−0.32, 0.36)	0.910
At Week 24	−2.20 (0.12)	−2.63 (0.12)	0.44 (0.10, 0.77)	0.011
2‐h PPG (mmol/L)				
At Week 12	−4.34 (0.22)	−4.70 (0.22)	0.36 (−0.24, 0.97)	0.236
At Week 24	−3.52 (0.23)	−4.11 (0.23)	0.59 (−0.03, 1.22)	0.064

*Note:* (1) Baseline is defined as the last non‐missing value before the first‐dose administration; (2) descriptive analysis was based on the actual observed values; (3) statistical analysis was performed using ANCOVA with the change from baseline in the HbA1c level at Week 12 as the dependent variable and the baseline HbA1c level, the stratification factor sex, and the grouping as the independent variables; (4) statistical analysis was performed using ANCOVA with the change from baseline in body weight at Week 12 and Week 24 as the dependent variable and the baseline body weight, stratification factors (sex and HbA1c level [< 8.5% and ≥ 8.5%]) and the grouping as the independent variables; (5) statistical analysis was performed using ANCOVA with the change from baseline in the FPG level at Week 12 and Week 24 as the dependent variable, and the baseline FPG level, stratification factors (sex and HbA1c level [< 8.5% and ≥ 8.5%]) and the grouping as the independent variables; (6) statistical analysis was performed using ANCOVA with the change from baseline in the 2‐h PPG level at Week 12 and Week 24 as the dependent variable and the baseline 2‐h PPG level, stratification factors (sex and HbA1c level [< 8.5% and ≥ 8.5%]) and the grouping as the independent variables.

#### 3.2.2 Body Weight Endpoint

As shown in Table 6, at Week 12, the mean changes in body weight from baseline to Week 12 in the LY05008 and dulaglutide groups were −2.01 and −1.71 kg, respectively. At Week 24, the mean changes in body weight from baseline in the LY05008 group and dulaglutide group were −2.68 and −2.42 kg, respectively. Body weight reduction from baseline to Week 12 and Week 24 was comparable between the two treatment groups.

### 3.3 Safety

A total of 207 patients (93.2%) with 1133 AEs in the LY05008 group and 202 patients (92.7%) with 1119 AEs in the dulaglutide group during the study are summarized in Table 7. A total of 202 patients (91%) in the LY05008 group and 196 patients (89.9%) in the dulaglutide group experienced 1046 and 1024 TEAEs, respectively. A total of 128 patients (57.7%) were reported to have 493 TEAEs in the LY05008 group, which were deemed study drug‐related TEAEs. Similarly, 134 patients (61.5%) in the dulaglutide group had 528 study drug‐related TEAEs. Most study drug‐related TEAEs were mild or moderate in severity in both groups, and no severe TEAEs were observed in the LY05008 group. However, 3 patients (1.4%) with four severe TEAEs, which were not considered drug related, were included in the dulaglutide group.

**TABLE 7:** Summary of all adverse events—FAS.

	LY05008 group (*N* = 222)		Dulaglutide group (*N* = 218)		Total (*N* = 440)	
	*n* (%)	Events	*n* (%)	Events	*n* (%)	Events
AEs	207 (93.2)	1133	202 (92.7)	1119	409 (93.0)	2252
TEAEs	202 (91.0)	1046	196 (89.9)	1024	398 (90.5)	2070
Study drug‐related TEAEs	128 (57.7)	493	134 (61.5)	528	262 (59.5)	1021
	50 (22.5)	196	51 (23.4)	125	101 (23.0)	321
Severe TEAEs	0	0	3 (1.4)	4	3 (0.7)	4
Severe study drug‐related TEAEs	0	0	0	0	0	0
AESIs	0	0	0	0	0	0
Patients who withdrew from the study due to TEAEs	4 (1.8)	8	7 (3.2)	13	11 (2.5)	21
Patients who withdrew l from the study due to study drug‐related TEAEs	4 (1.8)	8	6 (2.8)	10	10 (2.3)	18
SAEs	9 (4.1)	17	8 (3.7)	10	17 (3.9)	27
Study drug‐related SAEs	1 (0.5)	1	1 (0.5)	1	2 (0.5)	2
TEAEs leading to treatment interruption	4 (1.8)	6	1 (0.5)	2	5 (1.1)	8
Study drug‐related TEAEs leading to treatment interruption	4 (1.8)	6	1 (0.5)	1	5 (1.1)	7
TEAEs leading to permanent discontinuation of the drug	4 (1.8)	9	11 (5.0)	23	15 (3.4)	32
Study drug‐related TEAEs leading to permanent discontinuation of the drug	4 (1.8)	9	10 (4.6)	20	14 (3.2)	29
TEAEs leading to death	0	0	0	0	0	0
Study drug‐related TEAEs leading to death	0	0	0	0	0	0

*Note: N* denotes the total number of patients in the respective treatment group; *n* represents the number of patients with non‐missing values within a specific category. The percentages were based on the total number of patients in the specified treatment group. AEs were coded via MedDRA 26.1.

As shown in Tables 7, 8, 9 study drug‐related TEAEs leading to patient withdrawal occurred in four patients (1.8%) and six patients (2.8%) in the LY05008 group and dulaglutide group, respectively. Treatment interruption occurred in four patients (1.8%) with six TEAEs in the LY05008 group, which were all considered drug‐related TEAEs. Compared with the LY05008 group, one TEAE in the dulaglutide group led to treatment interruption in one patient (0.5%), which was regarded as a study drug‐related TEAE. Nine and twenty TEAEs occurred in 4 patients (1.8%) and 10 patients (4.6%) in the LY05008 group and dulaglutide group, respectively, leading to the permanent discontinuation of the study drug; these AEs were deemed study drug‐related TEAEs.

The common TEAEs reported in ≥ 10% of patients treated with LY05008 or dulaglutide were similar between the two groups (Table 8). Of these TEAEs, decreased appetite (21.6% vs. 28.4%), diarrhea (18.9% vs. 17.4%), nausea (12.6% vs. 15.6%) and vomiting (10.4% vs. 7.3%) were considered study drug‐related TEAEs. Most TEAEs and study drug‐related TEAEs were recovered in both groups (Table 9).

**TABLE 8:** Summary of treatment‐emergent adverse events (occurring in ≥ 10% of patients treated with LY05008 or dulaglutide)—FAS.

System organ class/preferred term	LY05008 group (*N* = 222)		Dulaglutide group (*N* = 218)		Total (*N* = 440)	
	*n* (%)	Events	*n* (%)	Events	*n* (%)	Events
At least experiencing one TEAE	202 (91.0)	1046	196 (89.9)	1024	398 (90.5)	2070
Metabolism and nutrition disorders	107 (48.2)	192	118 (54.1)	215	225 (51.1)	407
Decreased appetite	48 (21.6)	78	62 (28.4)	85	110 (25.0)	163
Hyperuricemia	39 (17.6)	50	42 (19.3)	49	81 (18.4)	99
Gastrointestinal disorders	97 (43.7)	317	91 (41.7)	303	188 (42.7)	620
Diarrhea	43 (19.4)	92	42 (19.3)	84	85 (19.3)	176
Nausea	28 (12.6)	46	34 (15.6)	65	62 (14.1)	111
Vomiting	23 (10.4)	42	19 (8.7)	26	42 (9.5)	68
Infections and infestations	76 (34.2)	102	91 (41.7)	131	167 (38.0)	233
Upper respiratory tract infection	39 (17.6)	41	43 (19.7)	49	82 (18.6)	90
Urinary tract infection	25 (11.3)	30	31 (14.2)	39	56 (12.7)	69
Investigations	82 (36.9)	196	69 (31.7)	135	151 (34.3)	331
Cardiac disorders	37 (16.7)	52	40 (18.3)	50	77 (17.5)	102
General disorders and administration site conditions	22 (9.9)	33	30 (13.8)	44	52 (11.8)	77

*Note: N* denotes the total number of patients in the respective treatment group; *n* represents the number of patients with non‐missing values within a specific category. The percentages were based on the total number of patients in the specified treatment group. TEAEs were coded via MedDRA 26.1.

**TABLE 9:** Summary of study drug‐related treatment‐emergent adverse events (occurring in ≥ 10% of patients treated with LY05008 or dulaglutide) in the FAS.

System organ class/preferred term	LY05008 group (*N* = 222)		Dulaglutide group (*N* = 218)		Total (*N* = 440)	
	*n* (%)	Events	*n* (%)	Events	*n* (%)	Events
At least experiencing one study drug‐related TEAE	128 (57.7)	493	134 (61.5)	528	262 (59.5)	1021
Gastrointestinal disorders	93 (41.9)	298	86 (39.4)	286	179 (40.7)	584
Diarrhea	42 (18.9)	89	38 (17.4)	78	80 (18.2)	167
Nausea	28 (12.6)	46	34 (15.6)	65	62 (14.1)	111
Vomiting	23 (10.4)	42	16 (7.3)	22	39 (8.9)	64
Metabolism and nutrition disorders	56 (25.2)	88	69 (31.7)	101	125 (28.4)	189
Decreased appetite	48 (21.6)	78	62 (28.4)	85	110 (25.0)	163
Investigations	25 (11.3)	46	21 (9.6)	43	46 (10.5)	89
General disorders and administration site conditions	12 (5.4)	21	22 (10.1)	31	34 (7.7)	52

*Note: N* denotes the total number of patients in the respective treatment group; *n* represents the number of patients with non‐missing values within a specific category. The percentages were based on the total number of patients in the specified treatment group. Study drug‐related TEAEs were coded using MedDRA 26.1.

In the LY05008 group and dulaglutide group, 3 patients (1.4%) and 10 patients (4.6%) were reported to experience 3 and 13 hypoglycemic events, respectively. Two patients (0.9%) with 2 hypoglycemic events and eight patients (3.7%) with 11 hypoglycemic events were attributed to LY05008 and dulaglutide administration, respectively. Severe hypoglycemia was not noted, nor were hypoglycemic events leading to patient withdrawal, treatment interruption, permanent discontinuation of the study drug, or death observed in either group during the study.

Seventeen SAEs occurred in nine patients (4.1%) and ten SAEs occurred in eight patients (3.7%) in the LY05008 and dulaglutide groups, respectively. Among these SAEs, one patient (0.5%) with one SAE was noted in each group (LY05008: gastric ulcer vs. dulaglutide: gastritis), which was regarded as a study drug‐related SAE. The patient in the LY05008 group who experienced a gastric ulcer recovered with treatment. In contrast, the patient who experienced gastritis attributed to dulaglutide did not improve following hospitalization, and the symptoms are still ongoing.

No notable differences in vital signs, physical examination results, laboratory test results, or 12‐lead ECG‐related TEAEs were observed between the LY05008 group and the dulaglutide group. No AEs of special interest or deaths were reported during the study in either group.

### 3.4 PK and Immunogenicity

**FIGURE 3:**
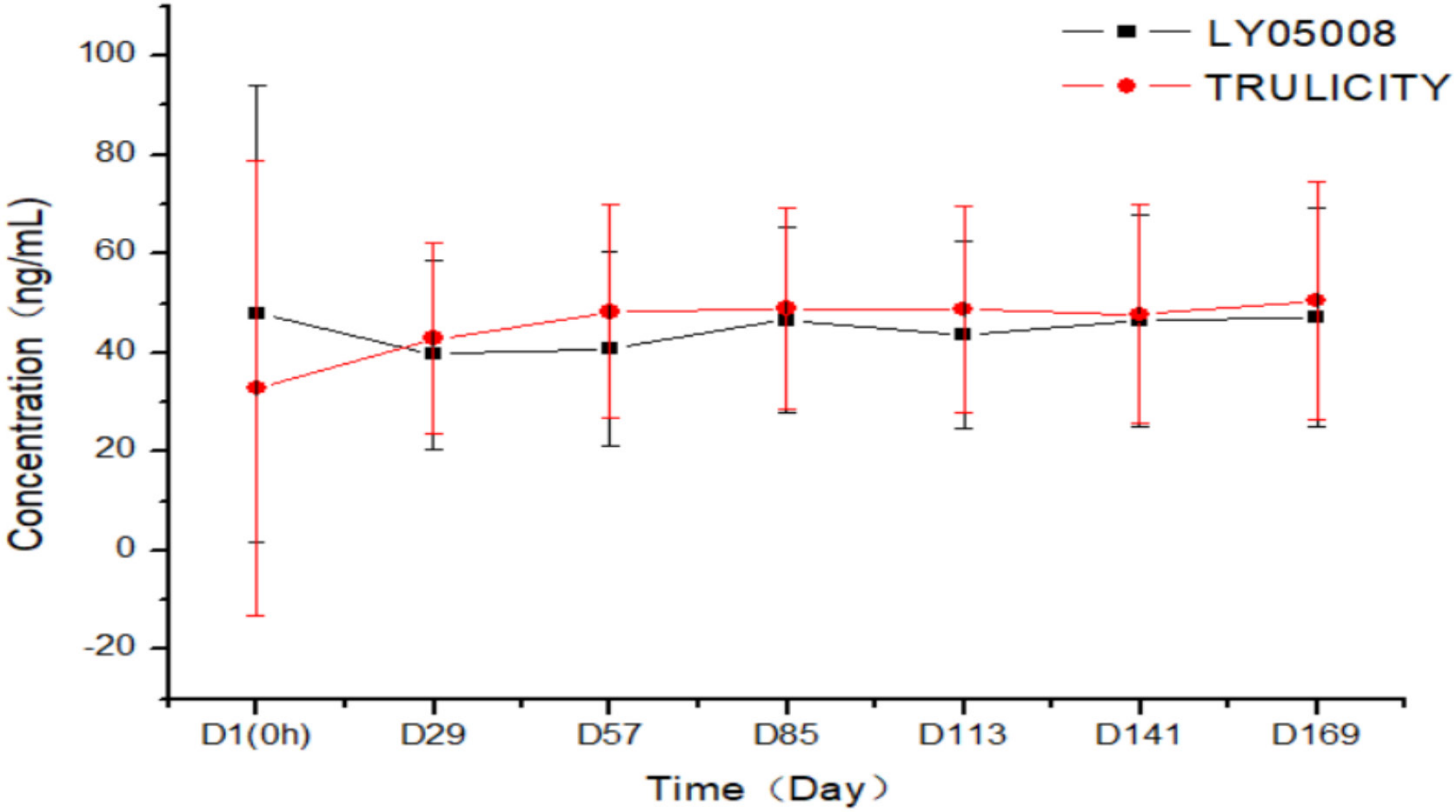
Mean plasma concentration‐time profiles of dulaglutide prior to and following the study drug administration in LY05008 group or dulaglutide group (linear scale). Black represents LY05008; red represents dulaglutide (Trulicity).

Per PKCS, three patients had measurable plasma concentrations prior to study drug administration on D1 (0 h) in the LY05008 group and dulaglutide group, respectively. Plasma concentrations of others (excluding three patients in each treatment group) were all below the lower limit of quantification (BLQ). The mean plasma concentrations on Days 29, 57, 85, 113, 141, and 169 in the LY05008 group and dulaglutide group prior to drug administration were 39.707 versus 42.789 ng/mL, 40.832 versus 48.269 ng/mL, 46.740 versus 48.905 ng/mL, 43.565 versus 48.755 ng/mL, 46.501 versus 47.736 ng/mL, and 47.121 versus 50.540 ng/mL, respectively, as shown in Figure 3.

Trough plasma concentrations at steady state between LY05008 group and dulaglutide group were comparable.

Among the SS, 35 patients (16.0%) in the LY05008 group and 53 patients (24.9%) in the dulaglutide group tested positive for anti‐drug antibodies (ADAs) during the study. All patients who were ADA positive in either group tested negative for neutralizing antibodies (Nab), except for one patient who tested Nab positive in the LY05008 group.

## 4 Discussion

ADA SOC 2024 recommended that GLP‐1 receptor agonists such as dulaglutide could be used as an initial therapy in most T2DM cases, including patients with HbA1C < 11% [15]. GLP‐1 receptor agonists not only contribute to HbA1C reduction to a greater extent, but promote weight loss and are associated with a low risk of hypoglycemia when compared with insulin. Additionally, GLP‐1 receptor agonists could provide cardiovascular benefits to patients with T2DM, which in turn reduces the risk of CVD death. Per Diabetes Mellitus: Efficacy Endpoints for Clinical Trials Investigating Antidiabetic Drugs and Biological Products Guidance for Industry, changes from baseline in A1C had been recognized and accepted as a primary endpoint for glycemic‐control indication in clinical trials [16]. Besides that, A1C is a valid surrogate endpoint for microvascular disease risk reduction.

The primary objective of this study was to compare the clinical efficacy of LY05008 with that of the reference drug dulaglutide following multiple subcutaneous injections. The result of this phase 3 trial has met the primary endpoint, the change from baseline in HbA1C level reduction to Week 24, with 95% CI falling within the range of (−0.4%, 0.4%). Based on a comprehensive review of dulaglutide clinical data focusing on the AWARD phase 3 clinical trial program, HbA1C reduction in response to 1.5 mg dulaglutide ranged from −0.78% to −1.64% versus LY05008 (−1.44%) depending on the baseline glycemic status and week of treatment [17]. In the LY05008 group and dulaglutide group, a comparable HbA1c level reduction from baseline was also observed at Week 12. Compared with patients receiving dulaglutide, patients receiving LY05008 had similar effects on the HbA1c level, FPG level, and 2‐h PPG level. Due to its mechanism of action, GLP‐1 receptor agonists lead to weight loss to some extent, and no significant differences in body weight reduction were noted between the groups. Furthermore, the results from the subgroup analysis stratified by sex and HbA1c level were congruent with those demonstrated in the primary analysis.

The secondary objective of this study was to demonstrate the comparable safety profiles, PKs, and immunogenicity between LY05008 and dulaglutide. The distribution and incidence of TEAEs between the two treatment groups were similar, indicating that these treatments are generally safe and well tolerated in Chinese adults with T2DM. The most common TEAEs (defined as ≥ 10%), including decreased appetite, diarrhea, nausea, and vomiting, were regarded as study drug‐related TEAEs, which were consistent with those reported in other clinical trials of reference drug labeling [5]. The majority of the study drug‐related TEAEs were deemed mild or moderate in severity in either group. Gastric ulcer in the LY05008 group and gastritis in the dulaglutide group were assessed as study drug‐related SAEs. The patient with gastric ulcer recovered after receiving the treatment intervention. The incidence of treatment discontinuation due to AEs was 6.1% in the 1.5 mg dulaglutide group in a 26‐week study. The most common adverse reactions leading to discontinuation were nausea (1.0%, 1.9%), diarrhea (0.5%, 0.6%), and vomiting (0.4%, 0.6%) for dulaglutide 0.75 and 1.5 mg, respectively, and were generally reported during the first 4 to 6 weeks [7]. The TEAEs in this study with respect to permanent discontinuation (3.4%) and treatment interruption (1.1%) were in line with those in the reference labeling.

During the study, all the hypoglycemic events that occurred in both groups were assessed as mild in severity and were followed by full recovery. Notably, no severe hypoglycemic events were detected in either group. Moreover, multiple injections of LY05008 over 24 weeks in Chinese T2DM patients had similar effects on vital signs, physical examination results, laboratory test results, and 12‐lead ECG data in the LY05008 group and dulaglutide group, which further demonstrated the totality of the safety profiles in both groups.

Measurable plasma concentrations were detected in three patients in the LY05008 group and the dulaglutide group, respectively, prior to study drug administration. Following the drug administration, plasma concentrations in both groups initially increased and ultimately reached the steady state at each blood sampling time point. The steady state plasma concentration was 39.707–47.121 ng/mL in the LY05008 group, while the plasma concentration at steady state in the dulaglutide group was 42.789–50.540 ng/mL. Trough plasma concentrations at steady state were comparable between the groups. None of the ADA‐positive patients had an impact on the plasma concentrations in either group. In the LY05008 group, no clinically significant differences were observed for those who were ADA positive; however, one patient tested Nab positive without an endogenous GLP‐1 cross‐reaction. In contrast, all patients in the dulaglutide group were Nab negative.

The results from a previous phase 1 trial demonstrated that the PK of LY05008 was similar to that of dulaglutide in healthy Chinese male subjects. Safety and immunogenicity profiles were similar between the groups [18]. A phase 1 trial was completed to further support the assessment of LY05008 in patients with T2DM in a phase 3 trial. A crossover design was not conducted to demonstrate if the HbA1c reduction would be similar between the groups for patients who had previously received dulaglutide but switched to LY05008 afterwards or vice versa.

## 5 Conclusion

In Chinese patients with T2DM following multiple subcutaneous injections, efficacy equivalence was achieved between LY05008 and dulaglutide with respect to change from baseline in HbA1C reduction to Week 24. Comparable effects in safety and immunogenicity profiles were observed between the two groups.

## Author Contributions

Li Liu, Zhifeng Cheng, Lianwei Wang, Lili Zhang, Shunbin Li, Shu Li, Shuguang Pang, Qifu Li, Fang Bian, Junling Gu, Jie Shen, Liujun Fu, Zhaoyang Zeng, and Lixin Guo worked on the drafting and critical revision of the manuscript. Baiping Sun, Yanyan Zhao, and Changlin Dou made the study concept and design. Li Liu, Zhifeng Cheng, Lianwei Wang, Lili Zhang, Shunbin Li, Shu Li, Shuguang Pang, Qifu Li, Fang Bian, Junling Gu, Jie Shen, Liujun Fu, Zhaoyang Zeng, and Lixin Guo worked on the study supervision and clinical data management. Baiping Sun, Yanyan Zhao, and Changlin Dou provided the guidance for implementation of the clinical study, and Lixin Guo, Li Liu, Zhaoyang Zeng, and Changlin Dou supported the medical monitoring.

## Conflicts of Interest

The authors declare no conflicts of interest.
